# Isolation of *Mycoplasma salivarium* in the empyema of a diabetic patient with deep neck infection and necrotizing mediastinitis: A case report

**DOI:** 10.1016/j.idcr.2023.e01915

**Published:** 2023-10-13

**Authors:** Ning-Chun Weng, Wen-Liang Yu

**Affiliations:** a Department of Internal Medicine, Chi Mei Medical Center, Tainan, Taiwan; b Department of Intensive Care Medicine, Chi Mei Medical Center, Tainan, Taiwan; c Department of Medicine, school of Medicine, College of Medicine, Taipei Medical University, Taipei, Taiwan

**Keywords:** *Mycoplasma spp*, *M. salivarium*, Empyema, PCR, MALDI-TOF-MS

## Abstract

*Mycoplasma species (spp.)* are predominantly found in the human oropharynx, and extracavity infections are rare. Conventional culture limitations hinder *Mycoplasma spp.* recovery, potentially causing overlooked infections. Molecular techniques reveal their roles in various infections. *Mycoplasma pneumoniae* causes pneumonia, while *Mycoplasma salivarium (M. salivarium)* in empyema is scarcely reported. We present a case of a 61-year-old man who suffered from tonsillitis, deep neck infection, necrotizing mediastinitis, and bilateral pleural infections. Mixed pathogens, mainly *M. salivarium*, were implicated.

## Introduction

*Mycoplasma spp* are unique bacteria that lack a cell wall and are the smallest free-living organism. *Mycoplasma spp.* preferentially isolated from human oropharynx, particularly from individuals with periodontal disease. The cases infected outsides of the oral cavity are rare. Because routine bacterial culture media and incubation conditions and times are not always adequate for the recovery of *Mycoplasma spp.*, it is likely that *Mycoplasma* infections often go undetected [1], [2]. The use of molecular techniques, especially polymerase chain reaction (PCR), has led to a better recognition of the role of such organisms in neurologic, cardiac, lung, digestive organs, dermatologic, and bone and joint infections [3]. *Mycoplasma pneumoniae* is a well-known cause of community-acquired pneumonia. However, there have been only a few reported cases implicating *M. salivarium* as a pathogen causing empyema, which is a condition characterized by pus accumulation in the pleural space. Thus, we describe a case of 61-year-old man with type 2 diabetes mellitus (T2DM), hypertension, and chronic kidney disease (CKD) suffered from tonsilitis, deep neck infection, descending necrotizing mediastinitis and bilateral pleural space infection caused by mixed pathogens involving *M. salivarium.*

## Case report

A 61-year-old man presented with several days of sore throat and left neck swelling not associated with fever, chills, or weight loss. The patient had the medical diseases of T2DM, hypertension, and CKD. His initial evaluation at another hospital showed deep neck infection and abscess formation in left masticator space after survey of the neck computed tomography (CT). He was intubated and transferred to the intensive care unit (ICU) of our hospital for additional care on June 19th, 2023 (day 0).

**Fig. 1:**
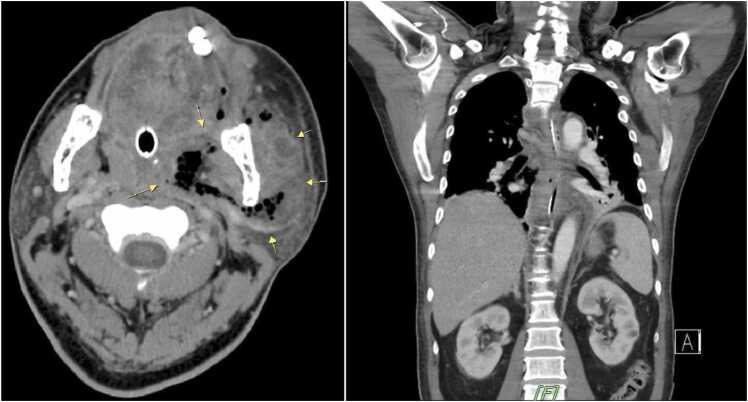
Initial CT scan image revealed abscess formation at left masticator space and palatine tonsil (yellow arrow), air-containing and fat stranding at middle and posterior mediastinum surrounding trachea, high suspected descending necrotizing mediastinitis(B), and bilateral basal lung atelectasis and empyema.

Upon arrival, he continued to require mechanical ventilation. Laboratory test from our emergency room disclosed band of 4.9 %, platelet count of 121,000/uL, glucose level of 383 mg/dL, azotemia with blood urea nitrogen of 23 mg/dL, high c-reactive protein (CRP) of 467 mg/L, HbA1c of 13.9 %, lactate level of 2.8 mmol/L, PO2/FiO2 of 298.2 mmHg, and ketone body of 2.3 mmol/L. Electrocardiography revealed sinus tachycardia with normal QTc of 445 miliseconds. Chest x-ray demonstrated increased infiltrations in the left lower lung field. Chest computed tomography revealed tonsil abscess, deep neck infection, and highly suspected descending necrotizing mediastinitis (Fig. 1). He was started on empirical antibiotic therapy of piperacillin sodium-tazobactam and clindamycin. A left cricopharyngeal myotomy with fasciectomy was performed on hospital day 1 and placed 2 combination waste vent (CWV) drainage. A bilateral video-assisted thorascopic (VATS) mediastinotomy with decortication was performed on hospital day 2 and placed 6 chest tubes drainage. The pathologic report indicates that the examined specimen consists of soft tissue fragments with extensive necrosis and purulent exudate from chest and fibroadipose tissue with mixed inflammatory infiltrate and focal fibrosis from neck. There was neither evidence of malignancy.

**Fig. 2:**
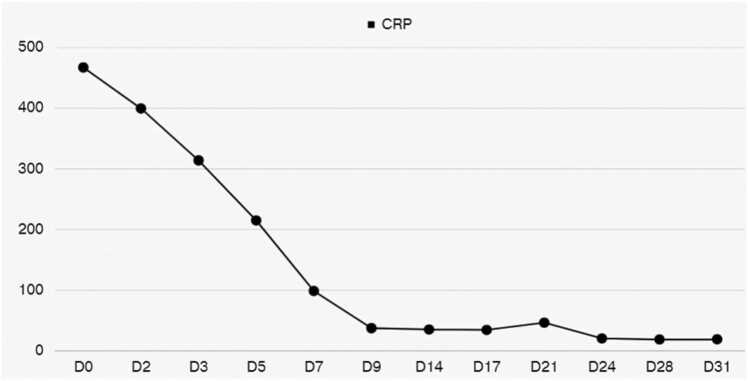
During the course of infection control, the CRP plasma concentrations exhibited a rapid decline. At day 0, the maximum CRP concentration peaked at 467 mg/l. However, by day 28, the CRP levels had significantly decreased, reaching a minimum concentration of 19 mg/l.

About 3 days later, *Streptococcus constellatus* was isolated from blood and pleural fluid. *M. salivarium* was additionally isolated from pleural fluid by Matrix-assisted laser desorption ionization–time of flight mass spectrometry (MALDI-TOF-MS). His infection treatment with piperacillin sodium-tazobactam and clindamycin was keeping and added levofloxacin. His diabetic ketoacidosis (DKA) resolved with medical treatment. On day 9 of hospitalization, he developed mild yellowish and cloudy discharge from CWVs and chest tube. We repeated neck and chest CT and reported residual abscess and pleural effusion. Fortunately, laboratory test revealed infection parameters improving (Fig. 2). After consulted with surgeons, esophagogastroduodenoscopy (EGD) was performed to excluded esophagus abscess or fistula formation.

**Fig. 3:**
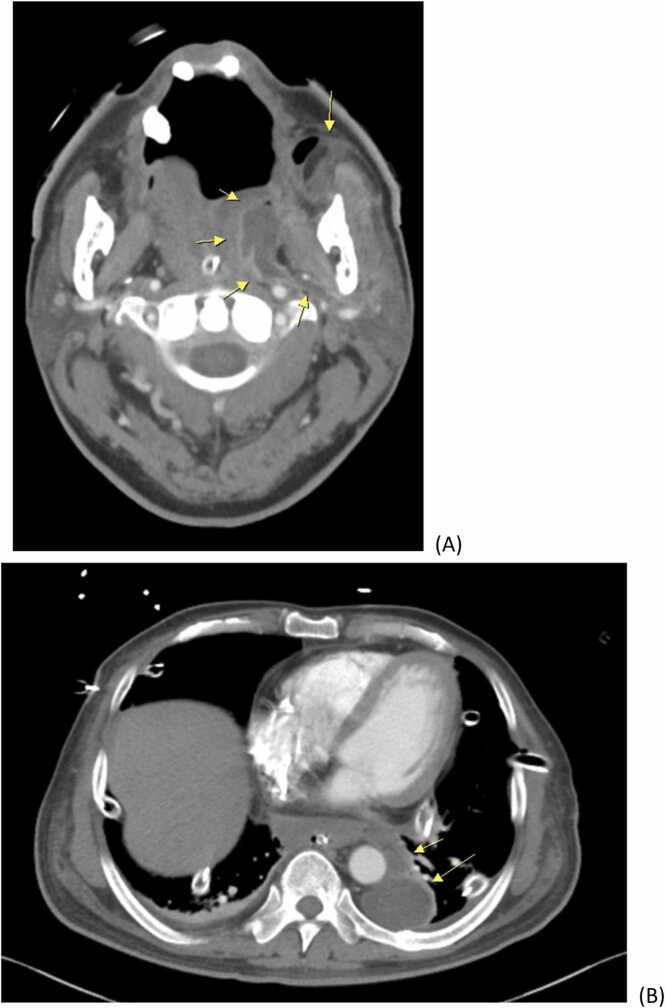
The repeated computed tomography scan was reported as below. Mild regression of residual abscess along left masseter muscle, submandibular region, palatine tonsil (yellow arrow), and mediastinum (A). Mild progressive bilateral lobulated complicated pleura effusion (yellow arrow) even adequate bilateral chest tube tips drainage function (B).

Despite appropriate antibiotic treatment, a follow-up CT scan 2 weeks later still showed mild progression of abscess accumulation at neck, mediastinum and pleura space (Fig. 3). As a result, the patient was arranged debridement operation again. After the second operation, he was weaning from ventilator and admitted to ordinary ward.

## Discussion

The development of severe inflammatory condition and unstable hemodynamic status is a big challenge for clinicians to resolve. *M. salivarium* is a difficulty to identify due to its small size and slow growth in routine culture media, which is why it is speculated that there is an underdiagnosis of infections causes [4], [5], [6], [7]. Successful culture requires specialized media and expertise, and cultures in appropriate media may nevertheless take several days or weeks to turn positive. Furthermore, back to this scenario, the patient with poor control for T2DM (HbA1C, 13.9 %) is considered an important risk group of immunosuppression. Culture and the patient’s poor health status were significant contributors to the progression from deep neck infection to descending necrotizing mediastinitis and empyema..

**Table 1:** Detection of *M. salivarium* and others pathogens in various sample over the time.

Data	Sample	Pathogen	Bacterial count	Growth in days
Day 0	^+^Blood	*S. constellatus*		5
Day 2	*Pleural fluid	*S. constellatus*	moderate	5
Day 2	*Pleural fluid	*M. salivarium*	heavy	5

^+^ The determination of the Minimum Inhibitory Concentration (MIC) of penicillin for the isolated *S. constellatus* was 0.125.

* The specimen was obtained by surgical intervention.

On the other hand, the management of a deep neck infection typically involves a combination of medical and surgical approaches to effectively manage the infection and prevent potential complications. Initially, broad-spectrum antibiotics are administered empirically to cover the most common pathogens from the oral and pharyngeal origin, including *Streptococcus pyogenes*, *Staphylococcus aureus*, and anaerobic bacteria. Once culture and sensitivity results become available, the antibiotic regimen can be adjusted to target the specific pathogens identified and their susceptibility patterns. In many cases, incision and drainage are required to drain the pus and infected material from the affected area. This helps to relieve pressure and reduce the spread of the infection. Besides, several risk factors for the development of severe deep neck infections and an affection of the mediastinum have been identified, such as age older than 55 years, cardiopulmonary comorbidities, nutritional status or especially diabetes mellitus [8], [9], [10], [11].

As for our patient, we prescribed levofloxacin as the chosen antibiotic after identifying *M. salivarium* in the pus sample. Currently, there is no established treatment guidelines for such infections. Due to the absence of a cell wall in all *Mycoplasma spp.*, they are inherently resistant to β-lactam antibiotics. A recent case reports performed demonstrates the main drugs showing in vitro susceptibility for *M. salivarium* are clindamycin, tetracycline, and moxifloxacin [5], [12], [14]. Acquired resistance to macrolides, lincosamides, fluoroquinolones, and tetracyclines has been documented in all of the *Mycoplasma spp.* known to be pathogenic in humans. Given the limited treatment options, careful monitoring of the patient's response to levofloxacin is essential [13], [14], [15].

The identification of the family *Mycoplasmataceae* infections is often challenging due to the small size of colonies and often inlaid in the agar, leading to difficulties in species- or genus-level identification through direct colony deposition [16]. Although genes encoding 16S rRNA have been commonly targeted for universal PCRs and subsequent analysis, individual PCR-based systems that were developed as species-specific tests might become cumbersome in a laboratory with over 100 *mycoplasmas* that are currently recognized as pathogens in humans and animals. In this context, MALDI-TOF-MS is considered to a powerful tool for the classification of a group of bacteria that are difficult to culture. It has proved to accurately distinguish species that are known to be closely related by MALDI-TOF-MS [17] . This method has shown to be sufficiently robust to be applicable with fastidious bacteria, such as anaerobic bacteria, *Legionella*, *Bartonella*, or *mycobacteria*. Additionally, it had generated a *mycoplasma* spectral database and more complete the database platform [16], [18].

In conclusion, *M. salivarium* infections can rarely contribute to empyema. Antibiotic therapy plays a vital role in the management of these infections, with fluoroquinolones and other antibiotics targeting *Mycoplasma spp.* being the treatment of choice. Accurate identification of the causative pathogen, facilitated by techniques like culture, MALDI-TOF-MS or molecular methods such as PCR, is essential for guiding appropriate antibiotic selection and optimizing patient outcomes. Further research is needed to explore the importance of early detection and precise sampling in establishing the correct diagnosis and preventing severe complications for the patient.

## Ethical approval

Written informed consent was obtained from the patient for the publication of this case report, including any associated images or clinical data.

## Consent

Written informed consent was obtained from the patient for the publication of this case report, including any associated images or clinical data. (The form was sign in Chinese, we could upload the picture if necessary).

## Funding

No conflicts of interest or funding sources were reported for this case report.

## Declaration of Competing Interest

The authors declare that they have no known competing financial interests or personal relationships that could have appeared to influence the work reported in this paper
